# The Protective Effect of Vitamin D on Dementia Risk in Hemodialysis Patients

**DOI:** 10.3390/life13081741

**Published:** 2023-08-13

**Authors:** Chih-Lang Lin, Wan-Ming Chen, An-Tzu Jao, Ben-Chang Shia, Szu-Yuan Wu

**Affiliations:** 1 Liver Research Unit, Department of Gastroenterology and Hepatology, Chang Gung Memorial Hospital, Keelung 204, Taiwan; 2 College of Medicine, Chang Gung University, Taoyuan 833, Taiwan; 3 Community Medicine Research Center, Chang Gung Memorial Hospital, Keelung Branch, Keelung 204, Taiwan; 4 Graduate Institute of Business Administration, College of Management, Fu Jen Catholic University, Taipei 242, Taiwan; 5 Artificial Intelligence Development Center, Fu Jen Catholic University, Taipei 242, Taiwan; 6 Big Data Center, Lo-Hsu Medical Foundation, Lotung Poh-Ai Hospital, Yilan 265, Taiwan; marine100120@gmail.com; 7 Department of Food Nutrition and Health Biotechnology, College of Medical and Health Science, Asia University, Taichung 413, Taiwan; 8 Division of Radiation Oncology, Lo-Hsu Medical Foundation, Lotung Poh-Ai Hospital, Yilan 265, Taiwan; 9 Department of Healthcare Administration, College of Medical and Health Science, Asia University, Taichung 413, Taiwan; 10 Cancer Center, Lo-Hsu Medical Foundation, Lotung Poh-Ai Hospital, Yilan 265, Taiwan; 11 Centers for Regional Anesthesia and Pain Medicine, Taipei Municipal Wan Fang Hospital, Taipei Medical University, Taipei 116, Taiwan

**Keywords:** Vitamin D, dose-dependent, dementia, risk, hemodialysis

## Abstract

**Simple Summary:**

This study investigated the effect of vitamin D supplementation on the risk of dementia in patients with end-stage renal disease on hemodialysis, which is a novel contribution to the literature. To our knowledge, this is the first study to examine the relationship between vitamin D supplementation and the risk of dementia in this patient population. The use of a 1:1 propensity score-matched cohort design adds robustness and reliability to the study findings. The results demonstrated that vitamin D supplementation at doses of ≥70 μg/week significantly reduced the risk of dementia in a dose-dependent manner, regardless of age. These findings add new and important information to the existing literature on the relationship between vitamin D and cognitive health in ESRD patients on hemodialysis, and suggest that vitamin D supplementation may be an effective preventive strategy for cognitive impairment in this vulnerable patient population.

**Abstract:**

Background: Patients with end-stage renal disease (ESRD) undergoing hemodialysis are at an elevated risk of developing dementia, potentially linked to the high prevalence of vitamin D deficiency in this population, which may contribute to cognitive impairment. Nevertheless, the impact of vitamin D supplementation on the risk of dementia in hemodialysis patients remains uncertain, necessitating further investigation to elucidate the potential benefits of vitamin D intervention in this vulnerable group. Methods: In this propensity-score-matched comparative cohort study, we sought to assess the impact of vitamin D supplementation on the occurrence of dementia in patients with end-stage renal disease (ESRD) undergoing hemodialysis. A total of 1424 patients were included and matched 1:1 using propensity scores. The study population was divided into two groups: those receiving vitamin D supplementation at a dose of ≥70 μg/week and those without any supplementation. The primary outcome of interest was the incidence of dementia. We calculated adjusted hazard ratios (aHRs) to examine the association between vitamin D supplementation and the risk of dementia while controlling for relevant covariates. Results: The adjusted hazard ratio (aHR) comparing vitamin D supplementation to no supplementation was 0.44 (95% CI 0.29–0.69; *p* < 0.0001), demonstrating a significant decrease in the risk of dementia associated with vitamin D supplementation. The aHRs for vitamin D supplementation at different dose ranges (70–105, 106–350, 351–1000, and >1000 μg/week) were 0.51, 0.49, 0.43, and 0.41, respectively (*p* for trend < 0.0001). These findings suggest a potential dose-dependent relationship between vitamin D supplementation and the reduction of dementia risk. Conclusions: In our study, we found that vitamin D supplementation at doses of ≥70 μg/week significantly reduced the risk of dementia in patients with end-stage renal disease (ESRD) undergoing hemodialysis. Furthermore, our results indicated a dose-dependent effect, with higher doses of supplementation correlating with a greater reduction in dementia risk. These findings underscore the potential of vitamin D supplementation as a preventive approach for cognitive impairment in this high-risk population.

## 1. Introduction

In Taiwan, the prevalence of chronic kidney disease (CKD) is estimated to be around 11.9%, with the disease affecting more than 2·5 million people [1]. Taiwan boasts the highest incidence and prevalence of end-stage renal disease (ESRD) globally, with over 90% of dialysis in Taiwan being hemodialysis [1,2,3,4]. Renal dysfunction in older adults is linked to a heightened risk of cognitive decline and dementia, possibly due to shared risk factors for vascular diseases, such as coronary heart disease, diabetes, and hypertension [5,6]. Fukunishi et al. [7] revealed that older adults on hemodialysis had a 7.4-fold higher annual incidence of dementia compared to older adults in the general population. These findings underscore the elevated risk of dementia among individuals with ESRD undergoing hemodialysis in Taiwan [3,4], and interventions, such as nutritional support, exercise, and medical management, may be necessary for the prevention, early detection, and attenuation of dementia [5,6,7,8].

Dementia is a devastating neurological condition that results in cognitive decline and impairments in daily functioning [9]. The global cost of caring for individuals with dementia is staggering, with estimates suggesting that in 2022 alone, USD 321 billion will be spent on healthcare, long-term care, and hospice services for those with the disease [10]. As the population ages and the number of individuals with dementia continues to rise, by 2060, projections suggest that the population of individuals affected by the condition is expected to reach 13.8 million, emphasizing the growing magnitude of the issue [10]. In order to tackle this urgent public health concern, it is imperative to identify modifiable risk factors for dementia and implement targeted interventions to mitigate the incidence and economic impact of the disease.

The association between vitamin D supplementation and the risk of dementia remains uncertain, necessitating further investigation and clarification. Prior research has provided evidence indicating an association between vitamin D deficiency and an elevated risk of dementia [11,12,13,14,15,16]. In 2022, a study found that vitamin D supplementation may exacerbate dementia progression, but this study did not consider factors such as vitamin D dosage, the severity of underlying diseases, or comorbidities associated with dementia, or adjust for competing mortality [17]. In order to better understand this relationship, we performed a comparative propensity score matching (PSM) analysis to investigate the impact of vitamin D on the risk of dementia in patients with ESRD who are undergoing hemodialysis [7,18]. Prior studies have firmly established the association between vitamin D deficiency and an elevated risk of developing dementia [7,18]. Patients with ESRD undergoing hemodialysis face a significantly higher risk of developing dementia, potentially linked to the high prevalence of vitamin D deficiency in this population, which may contribute to cognitive impairment [19,20,21]. While prior research has shown an association between vitamin D deficiency and an elevated risk of dementia [11,12,13,14,15,16], the relationship between vitamin D supplementation and dementia risk remains uncertain. Some studies have suggested that vitamin D supplementation may exacerbate dementia progression [17], but these studies lacked consideration of dosage and comorbidities associated with dementia. To address this uncertainty, our study utilized a comparative propensity-score matching analysis to investigate the impact of vitamin D on dementia risk in ESRD patients undergoing hemodialysis, aiming to provide more insights into this relationship.

## 2. Patients and Methods

### 2.1. Study Cohort

We retrieved data from the National Health Insurance Research Database (NHIRD) of Taiwan, encompassing the period from January 2004 to December 2020. The NHIRD contains comprehensive claims data from beneficiaries enrolled in the National Health Insurance program [22]. The NHIRD ensures the privacy and confidentiality of individuals by encrypting all data. Within this database, we accessed detailed outpatient and inpatient claims data, which encompassed patients’ identification numbers, birth dates, sex, diagnostic codes according to the International Classification of Diseases, Ninth Revision, Clinical Modification (ICD-9-CM), treatment details, medical costs, as well as dates of hospital admission, discharge, and death [22,23,24,25,26]. Our protocols were thoroughly reviewed and approved by the Institutional Review Board of Taipei Medical University (Approval No. 202212119).

### 2.2. Patient Selection

Patients aged 40 years or older who had undergone hemodialysis were included in this study. The index date was defined as the date of hemodialysis initiation. Exclusion criteria included a history of dementia prior to or within 1 year after the index date, as well as patients who died before 31 December 2020. Vitamin D supplementation was defined as the consumption of supplements containing vitamin D on most days, with a weekly dosage of ≥70 μg. Patients on hemodialysis who were prescribed ≥70 μg/week of vitamin D for more than 1 year constituted the treatment group, and those who were not prescribed vitamin D constituted the control group.

This study’s exclusion criteria were designed to ensure a specific and homogeneous study population. Firstly, patients with a history of dementia before or within 1 year after the index date were excluded to focus on incident cases of dementia rather than pre-existing conditions. This allowed for a clearer examination of the association between vitamin D supplementation and the risk of developing dementia. Secondly, patients who died before 31 December 2020 were excluded from this study. This exclusion aimed to avoid survival bias, as including patients who died during the study period could skew the results and lead to an inaccurate estimation of dementia risk. Additionally, individuals with any neurological disorders, such as Alzheimer’s and Parkinson’s disease, were excluded. This decision aimed to eliminate potential confounding factors that could influence this study’s outcomes and focus solely on the effect of vitamin D supplementation on dementia risk in hemodialysis patients. Furthermore, patients who discontinued hemodialysis treatment before 31 December 2020 were excluded from this study to minimize the impact of competing mortality. Discontinuation of hemodialysis could significantly affect the patient’s health status and lead to early mortality, introducing bias into the results. The defined daily dose (DDD) of vitamin D was converted into micrograms per week for ease of calculation and to standardize the measurement. Patients taking less than 70 μg/week of vitamin D were excluded from this study to ensure that the analysis focused on patients with consistent and significant vitamin D supplementation, rather than those with occasional or minimal intake.

The primary endpoint of this study was the occurrence of all-cause dementia. Dementia was defined based on specific ICD-9 cm codes (290.0, 290.1, 290.2, 290.3, 290.4, 294.1, or 331.0) recorded by board-certified psychiatrists or neurologists in accordance with the diagnostic criteria outlined in the Diagnostic and Statistical Manual of Mental Disorders, Fifth Edition (DSM-V). To ensure accurate identification of dementia cases, we required a minimum of three outpatient records or one hospital admission with a diagnostic code issued by a qualified psychiatrist or neurologist. It should be noted that our study employed a stricter definition of dementia compared to previous studies utilizing NHIRD data [27,28,29]. The NHIRD serves as a valuable population-level data source, offering real-world evidence that can inform clinical decision-making and healthcare policy development. Its comprehensive nature allows for the analysis of large patient cohorts, enabling robust evaluations of treatment outcomes, disease patterns, and healthcare utilization. The utilization of NHIRD data in our study provides a representative overview of the population, enhancing the generalizability and applicability of our findings to clinical practice and healthcare planning [30,31,32]. Continual efforts to validate diagnostic codes and refine methodological approaches have enhanced the reliability and validity of studies utilizing the NHIRD. The NHIRD has demonstrated high accuracy in diagnosing various diseases, making it a reliable resource for population-based research. These advancements in data quality and research methodologies have strengthened the credibility of NHIRD studies, providing valuable insights into disease epidemiology, treatment outcomes, and healthcare utilization at a population level [30,31,32].

### 2.3. PSM and Covariates

Patients prescribed vitamin D supplementation were identified from the National Health Insurance Research Database (NHIRD), and 1:1 propensity score matching (PSM) was performed to select patients not prescribed vitamin D supplementation from the remaining eligible database population. To account for potential confounders, a time-varying Cox proportional-hazards model was employed, taking into consideration the interval between the start of hemodialysis and a dementia diagnosis. A greedy method with a caliper size of 0.2 was used to match participants in a 1:1 ratio based on sex, age, income level, hospital type, and dementia-related comorbidities [33]. Comorbidities were determined based on primary ICD-9 cm codes from inpatient records, with patients requiring the corresponding code for ≥2 outpatient visits within 1 year to be considered as having the comorbidity. The analysis included comorbidities documented within 1 year before and after the index date. PSM is a widely used technique in clinical research to balance covariate distributions between treatment groups, while the standardized mean difference is commonly used to compare covariate distributions.

### 2.4. Dementia Hazard Ratios

We utilized time-varying Cox regression models to estimate adjusted hazard ratios (aHRs) and 95% confidence intervals (CIs) in order to compare the risks of dementia in patients undergoing hemodialysis with and without vitamin D supplementation.

### 2.5. Dose-Dependent Vitamin D Supplementation and Dementia Risk

The analysis of vitamin D dosage was based on the defined daily dose (DDD), as defined by the World Health Organization. The DDD represents the average daily maintenance dose of a drug used for its primary indication in adults [34]. To facilitate calculations, the defined daily dose (DDD) was converted into micrograms per week. Patients with a DDD of zero were considered not to be taking vitamin D. Moreover, to estimate the dose-dependent effects in the Cox regression model, the patients receiving vitamin D supplementation were subdivided into four groups based on dosage: 70–105, 106–350, 351–1000, or >1000 μg/week.

### 2.6. Subgroup Analysis

We conducted a subgroup analysis using a time-varying multivariate Cox regression model to assess the association between dementia and age, while adjusting for the confounding factors mentioned earlier.

### 2.7. Statistical Analysis

Continuous variables were presented as mean ± standard deviation, as appropriate. We employed a time-varying Cox regression analysis to assess the risk of dementia. To account for clustering within matched sets, a robust sandwich estimator was utilized. Furthermore, a time-varying multivariate Cox regression model was employed to estimate HRs with 95% CIs and identify potential independent predictors of dementia. All statistical analyses were conducted using SAS version 9.4 (SAS Institute, Cary, NC, USA). The significance level was set at *p* < 0.05, determined by a two-tailed Wald test. The cumulative incidence of dementia was calculated using the Kaplan–Meier estimator, and a stratified log-rank test was employed to compare the incidence of dementia, stratified according to matched sets.

## 3. Results

### 3.1. Study Cohort

PSM resulted in a final cohort of 1424 patients on hemodialysis, evenly distributed between the treatment and control groups (N = 712 each). The baseline characteristics of the two groups, including sex, age, income level, hospital type, and dementia-related comorbidities, were well-balanced after PSM, as shown in Table 1. The crude incidence rate of dementia differed significantly between the treatment and control groups (*p* < 0.0001; Table 1). The baseline characteristics of the two groups were well-balanced after PSM, and the incidence rate of dementia differed significantly between the treatment and control groups.

### 3.2. Predictors of Dementia in Patients on Hemodialysis

The time-varying multivariate Cox regression analysis revealed a significantly lower risk of dementia in the treatment group compared to the control group (Table 2). Following propensity score matching, there were no significant differences in sex, income level, hospital type, or dementia-related comorbidities between the two groups. The aHR for the treatment group, compared to the control group, was 0.44 (95% CI 0.29–0.69; *p* < 0.0001). Subgroup analysis demonstrated that compared to the 40–50-year-old subgroup, the aHRs (95% CIs) for the 51–60-year-old, 61–70-year-old, and >70-year-old subgroups were 2.51 (1.59–7.76), 4.18 (3.85–9.40), and 6.76 (5.36–11.23), respectively (Table 2). Time-varying multivariate Cox regression analysis revealed a significantly lower risk of dementia in the treatment group compared to the control group. Subgroup analysis indicated that age had a significant influence on dementia risk, with older age groups showing higher aHRs.

### 3.3. Risk of Dementia by Vitamin D Dosage

We further observed a dose–response relationship between vitamin D dosage and the risk of dementia. Compared to the control group (0 μg/week), the adjusted hazard ratios (aHRs) for vitamin D dosages of 70–105 μg/week, 106–350 μg/week, 351–1000 μg/week, and >1000 μg/week were 0.51, 0.49, 0.43, and 0.41, respectively (*p* < 0.0001) (Table 3). There was a dose–response relationship between vitamin D dosage and the risk of dementia. Higher vitamin D dosages were associated with lower risks of dementia.

### 3.4. Kaplan–Meier Curves Dementia

**Figure 1:**
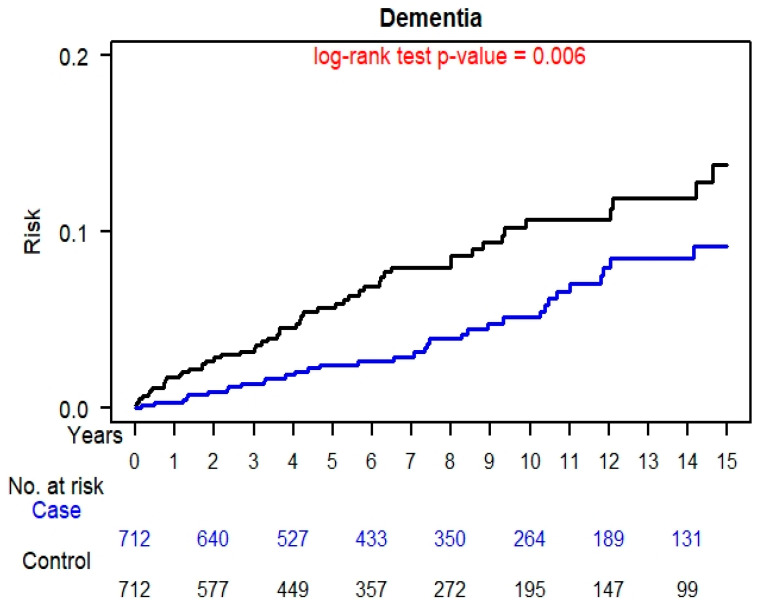
Cumulative incidence of dementia in patients with and without vitamin D supplementation.

The cumulative risks of dementia for the control and treatment groups are depicted in Figure 1. Notably, the cumulative risk of dementia was significantly higher in the control group compared to the treatment group (*p* = 0.0060). Kaplan–Meier curves depicted significantly lower cumulative risks of dementia in the treatment group compared to the control group.

### 3.5. Subgroup Analysis

**Figure 2:**
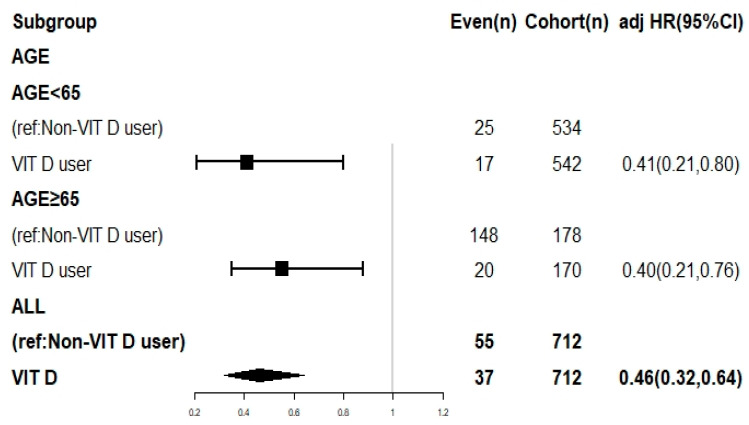
Subgroup analysis by age of dementia risk in patients with and without vitamin D supplementation. **Abbreviations:** CI, confidence interval; adj HR, adjusted hazard ratio.

To further explore the influence of age on dementia risk, a subgroup analysis was conducted by dividing the sample into two groups: aged ≥65 or <65 years. The analysis utilized a time-varying Cox regression model, and the findings are displayed in a forest plot depicted in Figure 2. Notably, in comparison to the treatment group, the control group exhibited significantly adjusted hazard ratios (aHRs) for dementia risk, indicating a significantly elevated risk of dementia in the absence of vitamin D supplementation, regardless of age. Subgroup analysis by age groups (<65 and ≥65 years) demonstrated that the treatment group consistently exhibited lower dementia risks compared to the control group, irrespective of age.

In conclusion, our study provides evidence supporting the protective effects of vitamin D supplementation on dementia risk in hemodialysis patients. The results indicate a significant reduction in dementia risk with vitamin D supplementation, and the protective effect appears to be dose-dependent. Subgroup analysis further confirms the benefits of vitamin D supplementation across different age groups.

### 3.6. Discussion

CKD represents a significant risk factor for dementia, and patients undergoing hemodialysis face an even higher risk. This heightened risk is evidenced by the annual incidence of dementia among older adults on hemodialysis, which is 7.4 times greater than that among older adults not receiving hemodialysis [7,35,36]. The potential benefits of nutritional intervention or medication in mitigating the risk of dementia among these patients are of considerable interest; however, the impact of vitamin D supplementation on dementia risk in this population remains uncertain [17,37]. Existing studies have yielded conflicting findings regarding the potential impact of vitamin D supplementation on the risk of dementia. While some studies have not observed a significant reduction in dementia risk with vitamin D supplementation, further investigation is needed to provide a clearer understanding of its effects [17,37], while others, including a retrospective observational study in 2022, have shown that vitamin D supplementation may even accelerate dementia progression [17]. However, these studies did not consider factors such as dosage, the severity of underlying diseases, or comorbidities related to dementia, or control for competing mortality [17]. The results of a preclinical study suggest that double-transgenic mice with certain gene variations may have lower vitamin D levels, but this effect was not observed in mice without these variations [17]. Specifically, the study found that mice with amyloid precursor protein and presenilin 1 gene variations, which are associated with early-onset Alzheimer’s disease but not all-cause dementia, had lower vitamin D levels. However, this effect was not observed in mice without a chimeric mouse/human amyloid precursor protein or variant human presenilin 1. These findings suggest a potential genetic specificity in the association between vitamin D levels and early-onset Alzheimer’s disease [17]. The main difference between our study and previous studies concerning a similar issue lies in the use of PSM to create a well-controlled comparative analysis. While existing studies have yielded conflicting findings regarding the impact of vitamin D supplementation on dementia risk [17,37], our study employed a rigorous PSM approach to minimize potential bias and provide compelling evidence of a significant reduction in the risk of all-cause dementia among patients with end-stage renal disease (ESRD) on hemodialysis receiving vitamin D supplementation. We observed a noteworthy adjusted hazard ratio (95% confidence interval) of 0.44 (0.29–0.69; *p* < 0.0001) for vitamin D supplementation, indicating a substantial protective effect. Notably, we identified a dose-dependent relationship, with higher doses of vitamin D associated with a progressively lower risk of dementia. Specifically, our findings revealed adjusted hazard ratios of 0.51, 0.49, 0.43, and 0.41 for vitamin D dosages of 70–105, 106–350, 351–1000, and >1000 μg/week, respectively (*p* < 0.0001). This study represents a significant advancement in the understanding of the impact of vitamin D supplementation on dementia risk in a high-risk population of patients with ESRD on hemodialysis.

Previous studies have consistently demonstrated an association between vitamin D deficiency and an increased risk of dementia in older adults [11]. Cross-sectional and case–control studies have shown that low vitamin D levels are associated with cognitive impairment, white matter abnormalities, and an elevated risk of all-cause dementia and Alzheimer’s disease [12,13,14]. Additionally, longitudinal studies have revealed an inverse relationship between vitamin D levels and cognitive decline over time [15]. However, the effectiveness of vitamin D supplementation in reducing the risk of dementia remains uncertain, with some low-quality reports suggesting no benefit [17,37]. The lack of controlled intervention studies hinders the identification of compelling reasons to prescribe vitamin D supplementation for preventing cognitive decline or dementia. Consequently, well-controlled intervention studies are warranted to confirm the dose-dependent effect of vitamin D supplementation in reducing dementia risk among hemodialysis patients [7,18].

Vitamin D deficiency has been implicated in the pathogenesis of dementia through several mechanisms. Studies suggest that vitamin D may play a role in the clearance of amyloid plaques in the brain [38,39], thereby reducing the risk of Alzheimer’s disease [40]. Additionally, vitamin D deficiency has been associated with vascular dysfunction and an increased risk of ischemic stroke [41], which are known contributors to cognitive decline and dementia. Furthermore, vitamin D deficiency has been linked to brain atrophy [42], which can contribute to the development of dementia. These findings highlight the potential mechanisms by which vitamin D deficiency may increase the risk of dementia. To date, no clinical studies have specifically examined the association between vitamin D and dementia risk in high-risk populations, such as patients with end-stage renal disease (ESRD) undergoing hemodialysis. Our study is the first well-controlled clinical investigation to assess the dose-dependent effects of vitamin D supplementation on dementia risk in this patient population. While our findings demonstrate a significant reduction in dementia risk with vitamin D supplementation, the underlying mechanisms responsible for these effects remain unclear and warrant further investigation.

In our study, we have not explored studies concerning the use of other plant extracts in relation to dementia risk. The focus of our study was specifically on vitamin D supplementation in hemodialysis patients. However, it is worth noting that previous research has investigated the association between other plant extracts and dementia risk. Some studies have explored the potential cognitive benefits of various herbal supplements, such as Ginkgo biloba, Bacopa monnieri, and Curcuma longa (turmeric), in reducing the risk of cognitive decline and dementia [43,44]. These studies often utilized observational designs or randomized controlled trials to assess the effects of these plant extracts on cognitive function [43,44]. Due to the fact that the aforementioned plant extracts are not covered by Taiwan National Health Insurance, we were unable to retrieve data on this category of plant extracts in our dataset. However, to offer a comprehensive understanding of the topic, it would be beneficial to conduct further investigation into the literature on plant extracts and their potential impact on dementia risk in the near future.

In this study, we employed a rigorous 1:1 PSM approach to minimize potential bias. Furthermore, we performed subgroup analyses by sex, age, income level, hospital type, and dementia-related comorbidities, and adjusted for relevant covariates to thoroughly investigate the impact of vitamin D supplementation on dementia risk. Our findings based on real-world data support the notion that vitamin D supplementation is associated with a reduced risk of dementia. However, further validation through large-scale randomized controlled trials is warranted to confirm these results definitively.

This study has several limitations that should be acknowledged. Firstly, the study participants consisted only of individuals of Asian descent, and caution should be exercised when generalizing the results to other populations. Secondly, the diagnosis of dementia relied on specific ICD-9 cm codes provided by professional psychiatrists or neurologists, which suggests accuracy but does not eliminate the possibility of misdiagnosis. The National Health Insurance system in Taiwan conducts audits and verifications to ensure the reliability of diagnoses recorded in the NHIRD. Nevertheless, a large-scale randomized controlled trial with carefully selected patients on hemodialysis and vitamin D supplementation may be necessary to provide more definitive evidence, although such trials are challenging to conduct in this patient population due to the limitations of tangible interventions [45]. Thirdly, as an observational study, we could not determine the reasons behind the decision to prescribe vitamin D supplementation. Fourthly, one limitation of our study is the lack of data on serum vitamin D concentrations. In clinical practice, the dosage of vitamin D supplementation is typically determined by monitoring serum calcium, phosphorus, and parathyroid hormone levels. Physicians are obliged to track these blood indicators when prescribing vitamin D. The patients’ condition must demonstrate improvements in these parameters for the physician to continue the prescription. Non-adherence to these regulations could lead to audits by the Taiwan National health insurance bureau and severe penalties, including a hundredfold increase in drug costs and exclusion of insurance coverage for that specific medication. Therefore, we believe that patients’ serum vitamin D concentrations generally experience some degree of improvement to justify the continuation of the medication prescription. Fifthly, our study did not directly measure estrogen levels in the NHIRD, as laboratory data is unavailable in NHIRD. However, in the context of hemodialysis patients, the hemodialysis procedure involves the clearance of metabolites and hormones, including estrogen, from the bloodstream. Consequently, the estrogen levels in hemodialysis patients are typically lower compared to those of individuals with normal kidney function [46]. Additionally, the matching process has already addressed age, a critical factor related to estrogen levels, within the study cohort. Considering that the study population consists of individuals with lower estrogen levels and age has been appropriately matched, we expect that the association between vitamin D supplementation and dementia risk in hemodialysis patients remains unaffected by estrogen levels. Sixthly, regarding adverse events in patients receiving 1000 mcg/week of vitamin D [47], our study did not specifically mention any observed adverse effects. However, it is important to note that vitamin D toxicity can occur with excessive doses, leading to hypercalcemia and related health issues [48]. Fortunately, our study cohort consisted of hemodialysis patients who undergo frequent treatments (three times a week at the hospital), which can help regulate blood calcium levels and minimize the risk of vitamin D toxicity (as nephrologists closely monitor electrolyte balance during hemodialysis sessions). The patients are closely monitored by kidney specialists, which can further mitigate potential adverse effects (three times a week). Due to the frequent hemodialysis sessions and close monitoring by nephrologists, the study cohort is less likely to experience kidney damage, bone problems, digestive system disturbances, and muscle and nerve problems associated with vitamin D toxicity. Lastly, the NHIRD lacked information on dietary habits, educational background, family history, and laboratory parameters, which could potentially influence the risk of dementia. Despite these limitations, the present study benefits from its use of a nationwide population-based registry with comprehensive baseline data and the ability to perform lifelong follow-up through linkage with the national cause-of-death register in Taiwan. Given the strong and statistically significant effects observed in this study, it is unlikely that the aforementioned limitations have significantly impacted our results.

## 4. Conclusions

In our study, we observed a significant dose-dependent reduction in the risk of dementia among patients on hemodialysis who received vitamin D supplementation. This effect was consistent across different age groups, indicating the potential effectiveness of vitamin D supplementation as an intervention for reducing dementia risk in patients with end-stage renal disease undergoing hemodialysis. However, further research is warranted to validate these findings and establish the optimal dosage and duration of vitamin D supplementation for preventing dementia in this specific population.

## Figures and Tables

**Table 1:** Characteristics of patients on hemodialysis with and without vitamin D supplementation (after propensity score matching).

	Without Vitamin D Supplementation		With Vitamin D Supplementation		
	N = 712		N = 712		*p* Value
	N	%	N	%	
**Sex**					0.6332
Female	364	51.12%	373	52.39%	
Male	348	48.88%	339	47.61%	
**Age (years)**					0.9735
40–50	284	39.89%	276	38.76%	
51–60	184	25.84%	190	26.69%	
61–70	139	19.52%	141	19.80%	
>70	105	14.75%	105	14.75%	
**Income level (NTD)**					0.8510
Low income	118	16.57%	127	17.84%	
≤20,000	90	12.64%	88	12.36%	
20,001–30,000	339	47.61%	325	45.65%	
>30,000	165	23.17%	172	24.16%	
**Hospital type**					0.9195
Medical center	164	23.03%	170	23.88%	
Regional hospital	449	63.06%	442	62.08%	
Clinic	99	13.90%	100	14.04%	
**Dementia-related comorbidities**					
Myocardial infarction	27	3.79%	24	3.37%	0.6688
Coronary heart disease	192	26.97%	197	27.67%	0.7662
Heart failure	151	21.21%	149	20.93%	0.8966
Peripheral vascular disease	68	9.55%	77	10.81%	0.4303
Chronic obstructive pulmonary disease	172	24.16%	191	26.83%	0.2480
Atrial fibrillation	13	1.83%	10	1.40%	0.5283
Hypertension	548	76.97%	549	77.11%	0.9498
Diabetes	274	38.48%	283	39.75%	0.6250
Dyslipidemia	285	40.03%	298	41.85%	0.4836
Traumatic head injury	11	1.54%	12	1.69%	0.8335
Depression	33	4.63%	43	6.04%	0.2384
Hearing loss	23	3.23%	24	3.37%	0.8821
Sleep apnea	4	0.56%	5	0.70%	0.7381
Peptic ulcer disease	293	41.15%	275	38.62%	0.3300
Chronic liver disease	229	32.16%	213	29.92%	0.3594
Rheumatic diseases	46	6.46%	46	6.46%	0.9999
Follow-up (years) Mean ± SD	8.87 ± 5.29		8.72 ± 5.05		0.5446
Follow-up (years) Median (IQR, Q1, Q3)	7.83 (2.66, 10.53)		7.93 (3.84, 12.32)		
**Outcomes**					
Dementia	72	10.11%	37	5.20%	0.0023

**Abbreviations**: SD, standard deviation; NTD, new Taiwan dollars; N, number; IQR, interquartile range; Q1, first quartile; Q3, third quartile.

**Table 2:** Results of Cox proportional hazards regression model of dementia risk in patients on hemodialysis with and without vitamin D supplementation.

	Crude HR	95% CI	*p* Value	Adjusted HR *	95% CI	*p* Value
**Vitamin D supplementation**						
Without vitamin D supplementation (ref.)	1.00			1.00		
With vitamin D supplementation	0.46	(0.32, 0.64)	<0.0001	0.44	(0.29, 0.69)	<0.0001
**Sex**						
Female (ref.)	1.00			1.00		
Male	0.87	(0.72, 1.05)	0.1499	0.68	(0.42, 1.09)	0.1075
**Age** (years)						
40–50 (ref.)	1.00			1.00		
51–60	2.28	(1.15, 5.01)	<0.0001	2.51	(1.59, 7.76)	0.0019
61–70	4.25	(3.27, 9.29)	<0.0001	4.18	(3.85, 9.40)	<0.0001
>70	6.31	(5.24, 13.39)	<0.0001	6.76	(5.36, 11.23)	<0.0001
**Income level (NTD)**						
Low income (ref.)	1.00			1.00		
≤20,000	0.84	(0.61, 1.14)	0.2542	0.50	(0.25, 1.19)	0.2464
20,001–30,000	0.91	(0.73, 1.13)	0.3865	0.48	(0.28, 1.22)	0.3274
>$30,000	0·62	(0.45, 1.26)	0.3554	0.44	(0.21, 1.14)	0.3470
**Hospital type**						
Clinic (ref.)	1.00			1.00		
Regional hospital	1.12	(0.90, 1.39)	0.301	1.04	(0.56, 1.94)	0.9039
Medical center	0.92	(0.67, 1.26)	0.6188	0.56	(0.26, 1.22)	0·1459
**Dementia-related comorbidities**						
Myocardial infarction	0.77	(0.52, 1.14)	0.1945	2.32	(0.98, 5.44)	0.0544
Coronary heart disease	1.02	(0.82, 1.25)	0.8801	1.32	(0.78, 2.22)	0.3060
Heart failure	1.10	(0.90, 1.34)	0.3744	0.77	(0.48, 1.26)	0.3026
Peripheral vascular disease	1.13	(0.89, 1.43)	0.3203	0.52	(0.25, 1.10)	0.0863
Chronic obstructive pulmonary disease	1.09	(0.9, 1.33)	0.3857	1.07	(0.64, 1.78)	0.8071
Atrial fibrillation	1.31	(0.84, 2.03)	0.2329	0.70	(0.81, 5.13)	0.7233
Hypertension	1.28	(0.95, 1.73)	0.1074	1.99	(0.96, 3.73)	0.3257
Diabetes	1.27	(1.03, 1.57)	0.0248	1.09	(0.66, 1.81)	0.7304
Dyslipidemia	0.98	(0.79, 1.2)	0.8116	1.78	(0.85, 3.01)	0.2321
Traumatic head injury	1.66	(0.92, 2.99)	0.0902	1.85	(0.85, 3.24)	0.2169
Depression	1.72	(1.32, 2.24)	<0.0001	1.03	(0.44, 2.39)	0.9426
Hearing loss	1.69	(1.25, 2.29)	0.0006	1.91	(0.66, 5.50)	0.2298
Sleep apnea	0.76	(0.52, 1.14)	0.0672	0.70	(0.58, 1.10)	0.9823
Peptic ulcer disease	1.14	(0.93, 1.39)	0.1987	0.98	(0.61, 1.55)	0.9217
Chronic liver disease	1.19	(0.98, 1.45)	0.0845	0.67	(0.40, 1.13)	0.1313
Rheumatic diseases	0.87	(0.61, 1.23)	0.4209	1.82	(0.83, 4.02)	0.1365

**Abbreviations:** NTD, new Taiwan dollars; CI, confidence interval; HR, hazard ratio; ref., reference group. * All covariates presented in Table 2 were adjusted for.

**Table 3:** Adjusted hazard ratios for dementia of vitamin D supplementation at various dosages.

	0 μg/Week		70–105 μg/Week		106–350 μg/Week		351–1000 Μg/Week		>1000 μg/Week		
**Dementia**	**N**	**%**	**N**	**%**	**N**	**%**	**N**	**%**	**N**	**%**	
No	640	89.89%	141	94.00%	165	94.83%	185	94.87%	184	95.34%	
Yes	72	10.11%	9	6.00%	9	5.17%	10	5.13%	9	4.66%	
											***p* for trend**
aHR * (95% CI)	1.00	ref.	0.51	(0.23, 0.92)	0.49	(0.26, 0.93)	0.43	(0.22, 0.84)	0.41	(0.24, 0.81)	<0.0001

**Abbreviations:** CI, confidence interval; aHR, adjusted hazard ratio; N, number; ref., reference group. * All covariates presented in Table 2 were adjusted for.

## Data Availability

The datasets supporting the study conclusions are included within this manuscript and its additional files.

## Abbreviations

HR: hazard ratio; aHR, adjusted hazard ratio; CI, confidence interval; ICD-9-CM, International Classification of Diseases, Ninth Revision, Clinical Modification; PSM, propensity-score matching; NHIRD, National Health Insurance Research Database; DDD, defined daily dose; ESRD, end-stage renal disease; CKD, chronic kidney disease; PMP, per million population; SMD, standardized mean difference.
